# Virtual Reality for Emergency Medicine Training in Medical School: Prospective, Large-Cohort Implementation Study

**DOI:** 10.2196/43649

**Published:** 2023-03-03

**Authors:** Moritz Mahling, Robert Wunderlich, Daniel Steiner, Eleonora Gorgati, Teresa Festl-Wietek, Anne Herrmann-Werner

**Affiliations:** 1 Tübingen Institute for Medical Education (TIME) Eberhard Karls University Tübingen Germany; 2 Department of Psychosomatic Medicine and Psychotherapy University Hospital Tübingen Tübingen Germany; 3 University Department of Anesthesiology and Intensive Care Medicine University Hospital Tübingen Eberhard Karls University Tübingen Germany

**Keywords:** emergency medicine, resuscitation, virtual reality, simulation, undergraduate medical education, Germany, medical education, virtual training, digital learning, medical student

## Abstract

**Background:**

Virtual reality (VR)–based simulation is being increasingly used to train medical students in emergency medicine. However, because the usefulness of VR may depend on various factors, the best practices for implementing this technology in the medical school curriculum are yet to be determined.

**Objective:**

The overall objective of our study was to assess the perceptions of a large cohort of students toward VR-based training and to identify the associations between these attitudes and individual factors, such as gender and age.

**Methods:**

The authors implemented a voluntary, VR-based teaching session in the emergency medicine course at the Medical Faculty in Tübingen, Germany. Fourth-year medical students were invited to participate on a voluntary basis. Afterward, we asked the students about their perceptions, collected data on individual factors, and assessed the test scores achieved by them in the VR-based assessment scenarios. We used ordinal regression analysis and linear mixed-effects analysis to detect the impact of individual factors on the questionnaire answers.

**Results:**

A total of 129 students participated in our study (mean age 24.7, SD 2.9 years; n=51, 39.8% male; n=77, 60.2% female). No student had previously used VR for learning, and only 4.7% (n=6) of the students had prior experience with VR. Most of the students agreed that VR can convey complex issues quickly (n=117, 91%), that VR is a useful addition to mannequin-based courses (n=114, 88%) or could even replace them (n=93, 72%), and that VR simulations should also be used for examinations (n=103, 80%). However, female students showed significantly less agreement with these statements. Most students perceived the VR scenario as realistic (n=69, 53%) and intuitive (n=62, 48%), with a relatively lower agreement for the latter among female respondents. We found high agreement among all participants (n=88, 69%) for immersion but strong disagreement (n=69, 54%) for empathy with the virtual patient. Only 3% (n=4) of the students felt confident regarding the medical content. Responses for the linguistic aspects of the scenario were largely mixed; however, most of the students were confident with the English language (not native) scenarios and disagreed that the scenario should be offered in their native language (female students agreed more strongly than male students). Most of the students would not have felt confident with the scenarios in a real-world context (n=69, 53%). Although physical symptoms during VR sessions were reported by 16% (n=21) of the respondents, this did not lead to the termination of the simulation. The regression analysis revealed that the final test scores were not influenced by gender, age, or prior experience in emergency medicine or with virtual reality.

**Conclusions:**

In this study, we observed a strong positive attitude in medical students toward VR-based teaching and assessment. However, this positivity was comparatively lower among female students, potentially indicating that gender differences need to be addressed when VR is implemented in the curriculum. Interestingly, gender, age, or prior experience did not influence the final test scores. Furthermore, confidence regarding the medical content was low, which suggests that the students may need further training in emergency medicine.

## Introduction

Emergency medicine is a clinical specialty that requires fast decision-making in scenarios that are usually complex and time sensitive, such as cardiopulmonary resuscitation (CPR) [1]. To impart the necessary skills, teachers currently use a variety of approaches, such as theoretical lessons, skill training, and simulation training with low- or high-fidelity simulators [2]. However, patient care in emergency medicine relies on various audiovisual stimuli and interpersonal interactions, which can be summarized under the term “situational awareness” [3]. As the training for these skills is best conducted in a simulated environment, virtual reality (VR) simulators are being increasingly used to facilitate the acquisition of such complex competencies [4].

VR technology can be grouped under the generic term extended reality and immerses the user in a completely virtualized world. To achieve the entire virtual illusion, special head-mounted displays are required. Furthermore, a special controller allows the user to interact with this VR. It provides an opportunity for experiential learning and improves contextualization [5], which are both important aspects in the acquisition of emergency medicine competencies. Emergency medicine training today mostly relies on physical simulators, but VR offers some opportunities that can benefit learning. First, although physical patient simulators are very realistic, VR can even increase the perceived reality and thus close a gap between what physical patient simulators can deliver and what physicians experience in real life [6]. Second, well-made high-fidelity simulation scenarios in real world are staff intensive, thus increasing the costs of the simulations. Once established, the only setup for a VR-based training session is the VR hardware, a room, and sometimes an operator, thereby lowering the obstacles for repeated training sessions. Third, emergency medicine covers some comparatively rare competencies (with CPR being among the most common “rare competencies”), again increasing the efforts to provide good training to all providers. The almost endless possibility to repeat even such rare scenarios is another advantage of VR-based emergency medicine training. Consequently, VR training has been successfully used for various training settings in emergency medicine, such as resuscitation training [7] and assessment [4]. A recent meta-analysis supports these findings, concluding that VR has had a positive impact on educational outcomes [8].

Despite its increasing use, the best practices for implementing VR in emergency medicine training for medical students are yet to be determined [4,6]. For such a VR-based teaching approach to be successful, several parameters need to be considered, including technical constraints, psychological aspects, and curricular integration [9]. Furthermore, it remains unclear if teaching and assessment using VR are suitable for all medical students. For example, research from real-life simulators has shown that gender can influence a student’s performance in a simulation [10,11]. A recent review highlighted this aspect as being important for further research on how VR can be modified to better serve training and assessment needs [4].

Therefore, to identify the factors that need to be addressed before VR is implemented in the medical curriculum, it is also important to adopt the perspective of the learner. Further, it is necessary to identify students who need special attention to ensure equal teaching success. Thus, we performed a large-scale assessment of learners’ perceptions of a VR simulation during a regular emergency medicine course. Furthermore, we collected data on demographics and assessed the impact of these variables on subjective and objective measures. The feasibility of such training, the additional value gained, the appreciation received from the students, and the occurrence of side effects were of particular interest.

## Methods

### Ethics Approval

The ethics committees of the Faculty of Medicine at Tübingen University Hospital approved the study (340/2021BO2). All methods were implemented in accordance with the Helsinki declaration. Participation in the study was voluntary.

### Study Design and Setting

A prospective, monocentric, observational study was conducted at Tübingen University, Germany, from June 2021 until April 2022. Data collection was conducted as a voluntary addition to the regular emergency medicine course, which was delivered by experienced teachers of the Department of Anaesthesiology.

The curricular emergency medicine course began with an inverted classroom unit consisting of a series of asynchronous theoretical web-based videos. The topics that were covered included (1) an overview of all relevant emergency medicine topics (eg, internal medicine, trauma, surgical, paediatric, gynaecological, neurological, psychiatric, and mass casualty) and (2) resuscitation, including a theoretical background and demonstrations of the relevant skills. The practical course itself consisted of 4 days of training, with a practical exam on resuscitation skills on the last day. During the waiting time between practical units, students were approached by a study assistant and informed about the VR study. Students who agreed to participate were familiarized with the VR setup and the simulation program before starting the simulation. Afterward, they completed a paper-based purpose-designed questionnaire.

### Recruitment

The studied population consisted of undergraduate medical students in their fourth year of training at the Medical Faculty in Tübingen, Germany. The VR session took place within the regular emergency medicine course over 1 semester; therefore, the 196 students who were enrolled in this class were potential participants in the study. Although participation in the class was mandatory, participation in the study was voluntary and was only possible after signing the informed consent forms. The only exclusion criterion was known epilepsy. The teachers were all experienced academic staff who were used to the teaching format and were randomly assigned to the student groups via the regular administrative assignment process. The VR session was supervised by a research assistant who had received specific training in the VR scenarios as well as the technical setup by the manufacturers of the VR equipment.

### Interventions

To familiarize the students with the general setup, we first introduced them to the hardware components (eg, VR head-mounted display, controller, and laptop); simulation software (Oxford Medical Simulation); and the potential scenarios, including specifications of their expected role. The Oxford Medical Simulation software was developed to train health care professionals in emergency patient management in a virtual simulated environment. It offers a variety of scenarios commonly encountered in a general emergency room. Among the scenarios offered, we chose 4 for training purposes according to the learning objectives of the course. The trainee, or the student in our case, was asked to take on the role of the emergency physician and had to interact with virtual persons (eg, patients and nurses) and equipment (eg, blood pressure measurement system and infusion) without real haptic feedback. The hardware included a head-mounted display, a hand-tracking device (Oculus Rift S; Facebook Inc), and a high-performance laptop (Alienware m15R3; Dell Computers).

To ensure familiarity with the VR simulation environment, the students were required to practice 4 emergency encounters in the virtual emergency room: (1) acute coronary syndrome, (2) sepsis, (3) stroke, and (4) exacerbated chronic obstructive pulmonary disease. Each scenario lasted for a maximum duration of 15 minutes. The trainees performed the role of the emergency physician and entered a patient room, where a patient and nurse were waiting. They had to perform the appropriate examinations, lab tests, invasive procedures, and treatments as well as ensure adequate communication with the patient, support staff, and emergency nurses in a timely manner. Afterward, students received the automatically generated results regarding their performance. Subsequently, they were required to undergo an assessment scenario on CPR that took place in the VR environment. The necessary procedures and actions were the same as those in the corresponding training scenario, with a maximum of 15 minutes allotted. The result of the assessment in terms of the percentage of correctly completed steps was noted, and the students were asked to complete the questionnaire. They were accompanied by a study assistant during the entire procedure, who would provide support in case of technical difficulties.

### Measurements and Outcomes

We developed a questionnaire to assess the perspectives of the learner on the curricular implementation of VR in emergency medicine as well as other aspects, such as physical symptoms, within one form (Textbox 1). The participating students filled in the questionnaire immediately after their assessment scenario. To enhance face validity, we applied the think-aloud technique, which was first tested by experts in the fields of emergency medicine and psychology. The questionnaire consisted of 13 statements that the students were asked to rank based on an anchored 5-point Likert scale (0=strongly disagree to 4=strongly agree). The items were related to features of the VR scenario, their own role in the simulation, suggestions for future implementation, and physical symptoms that they experienced. Furthermore, we collected data on general demographics; previous VR experience in different contexts, such as gaming or training; as well as the results of the assessment scenario.

**Questionnaire with statements to be ranked according to a 5-point Likert scale. ER: emergency room; VR: virtual reality.**

**Questionnaire “ER in VR” items**

Learning with VR is useful for conveying complex issues quickly.
Learning with VR is a useful addition to the practical face-to-face course with a resuscitation mannequin.
Practicing in VR should replace practicing on the resuscitation mannequin.
VR simulations should also be used for examinations.
The VR scenarios were realistic.
The VR scenarios and their handling were intuitive.
I could quickly put myself into the simulated situation.
I could empathically put myself in the situation of the virtual patient.
I felt confident in the scenario regarding medical content.
I felt confident in the scenario regarding linguistic aspects.
The VR simulation should be offered in German (not English).
I would have felt confident in the scenario in the real context (ie, with real patients).
(a) During the VR simulation I experienced physical symptoms (eg, dizziness, blurred vision, and image shifts) and (b) if yes, which symptoms?

### Statistical Analysis

All statistical analyses were performed using the programming language R [12]. We decided to treat the questionnaire answers on the Likert scale as not-normally distributed and thus treated them as ordinary distributed data. To assess the impact of gender and previous emergency medical services (EMS) experience, we performed an ordinal regression analysis using the *polr* function from the MASS (version 7.3-57, Modern Applied Statistics with S) package [13]. To detect a correlation between final Test Score and age, gender, previous experience with VR, as well as previous EMS experience, we performed a linear mixed-effects analysis using the *lme4* package in R [14]. We used age, gender, previous experience with VR, as well as previous EMS experience as fixed effect (without interaction) and an intercept for “term” as random effect. The *P* values were generated by a likelihood ratio test for the full model. Values that were <.05 were considered statistically significant.

## Results

### Characteristics of Study Subjects

Out of the total of 196 students who were attending the emergency medicine training as a part of their seventh semester of medical school, 129 agreed to participate in the study (response rate=65.8%). Among the respondents, 39.8% were male (n=51) and 60.2% (n=77) were female. The mean age was 24.7 (SD 2.9) years. Approximately 16.4% (n=21) had previously received vocational training in emergency services. While 43 students took part in the winter semester (2020 and 2021), 86 participated in the summer term in 2021. None of the students had previous experience in learning with VR, and only 4.7% (n=6) of the students had private experience with VR through video games.

### Questionnaire Results

**Figure 1:**
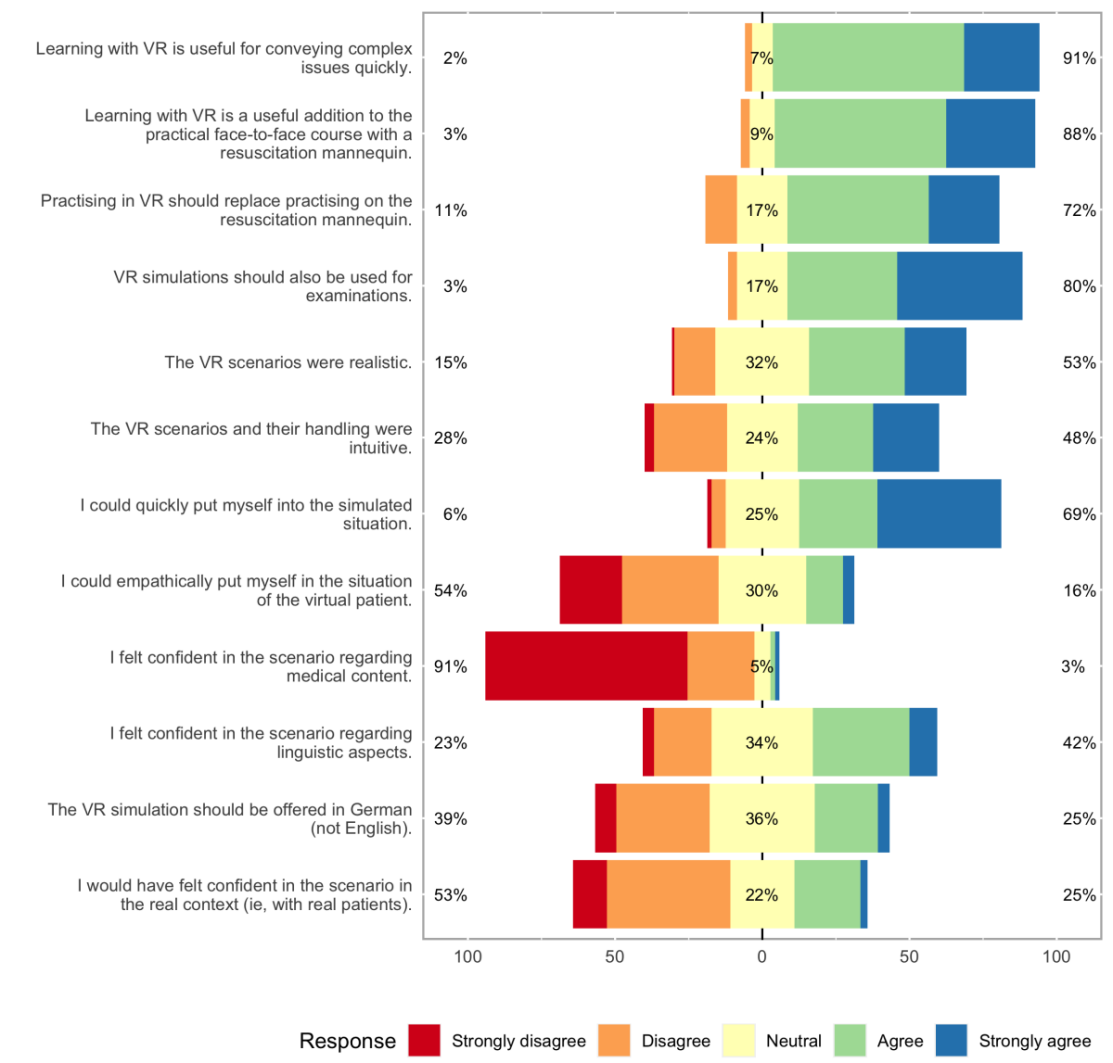
Likert plot of all questionnaire results for questions 1-12 (Q1-Q12). The percentages presented represent the sum of all participants that “strongly disagreed” and “disagreed” (values on the left), that were “neutral” (centered values), and that “agreed” and “strongly agreed” (values on the right). VR: virual reality.

The respondents showed strong agreement in their responses, with 91% (n=117) stating that VR is useful for conveying complex issues quickly (question 1 [Q1] in Table 1 and Figure 1). Similarly, 88% (n=114) agreed with the statement, “Learning with VR is a useful addition to the practical face-to-face course with a resuscitation mannikin” (Q2). Although most of the students (n=93, 72%,) thought that practicing in simulated VR environments should replace practicing on the resuscitation mannikin (Q3), 11% (n=14) disagreed with this statement. However, 80% (n=103) of the students stated that VR simulations should be used for examinations (Q4). Among the respondents, 53% (n=69) felt that the scenarios were realistic, while 15% (n=19) disagreed (Q5). Further, 48% (n=62) found the VR scenario and its handling (Q6) to be intuitive and 28% (n=36) disagreed, with 3% (n=4) disagreeing strongly. However, most of the students (69%, n=88) were able to quickly adapt to the simulated scenario (Q7). When asked if students could act empathically with the virtual patient (Q8), only 16% (n=21) agreed and 54% (n=69) disagreed. When asked for their confidence regarding the medical content of the scenario (Q9), 91% (n=117) of the students disagreed or strongly disagreed, and only 3% (n=4) agreed. Responses regarding the linguistic aspects of the scenarios (Q10) were mixed and most of the students (n=44, 34%) were neutral. Moreover, when asked if the scenario should be offered in German instead of English (Q11), 36% (n=45) were neutral. Approximately 53% (n=69) of the respondents did not feel confident in the scenario in the real context (Q12), while 25% (n=32) did.

**Table 1:** Detailed questionnaire results for questions 1-12 (Q1-Q12). The relative number of answers is indicated.

Item	Strongly disagree, n (%)	Disagree, n (%)	Neutral, n (%)	Agree, n (%)	Strongly agree, n (%)
1) Learning with VR^a^ is useful for conveying complex issues quickly.	0 (0)	3 (2.3)	9 (7)	84 (65.1)	33 (25.6)
2) Learning with VR is a useful addition to the practical face-to-face course with a resuscitation mannequin.	0 (0)	4 (3.1)	11 (8.5)	75 (58.1)	39 (30.2)
3) Practising in VR should replace practicing on the resuscitation mannequin.	0 (0)	14 (10.9)	22 (17.1)	62 (48.1)	31 (24)
4) VR simulations should also be used for examinations.	0 (0)	4 (3.1)	22 (17.1)	48 (37.2)	55 (42.6)
5) The VR scenarios were realistic.	1 (0.8)	18 (14)	41 (31.8)	42 (32.6)	27 (20.9)
6) The VR scenarios and their handling were intuitive.	4 (3.1)	32 (24.8)	31 (24.0)	33 (25.6)	29 (22.5)
7) I could quickly put myself into the simulated situation.	2 (1.6)	6 (4.7)	32 (25)	34 (26.6)	54 (42.2)
8) I could empathically put myself in the situation of the virtual patient.	27 (21.1)	42 (32.8)	38 (29.7)	16 (12.5)	5 (3.9)
9) I felt confident in the scenario regarding medical content.	88 (68.8)	29 (22.7)	7 (5.5)	2 (1.6)	2 (1.6)
10) I felt confident in the scenario regarding linguistic aspects.	5 (3.9)	25 (19.5)	44 (34.4)	42 (32.8)	12 (9.4)
11) The VR simulation should be offered in German (not English).	9 (7.1)	40 (31.7)	45 (35.7)	27 (21.4)	5 (4.0)
12) I would have felt confident in the scenario in the real context (ie, with real patients).	15 (11.6)	54 (41.9)	28 (21.7)	29 (22.5)	3 (2.3)

^a^VR: virtual reality.

### Impact of Gender on Questionnaire Answers

**Figure 2:**
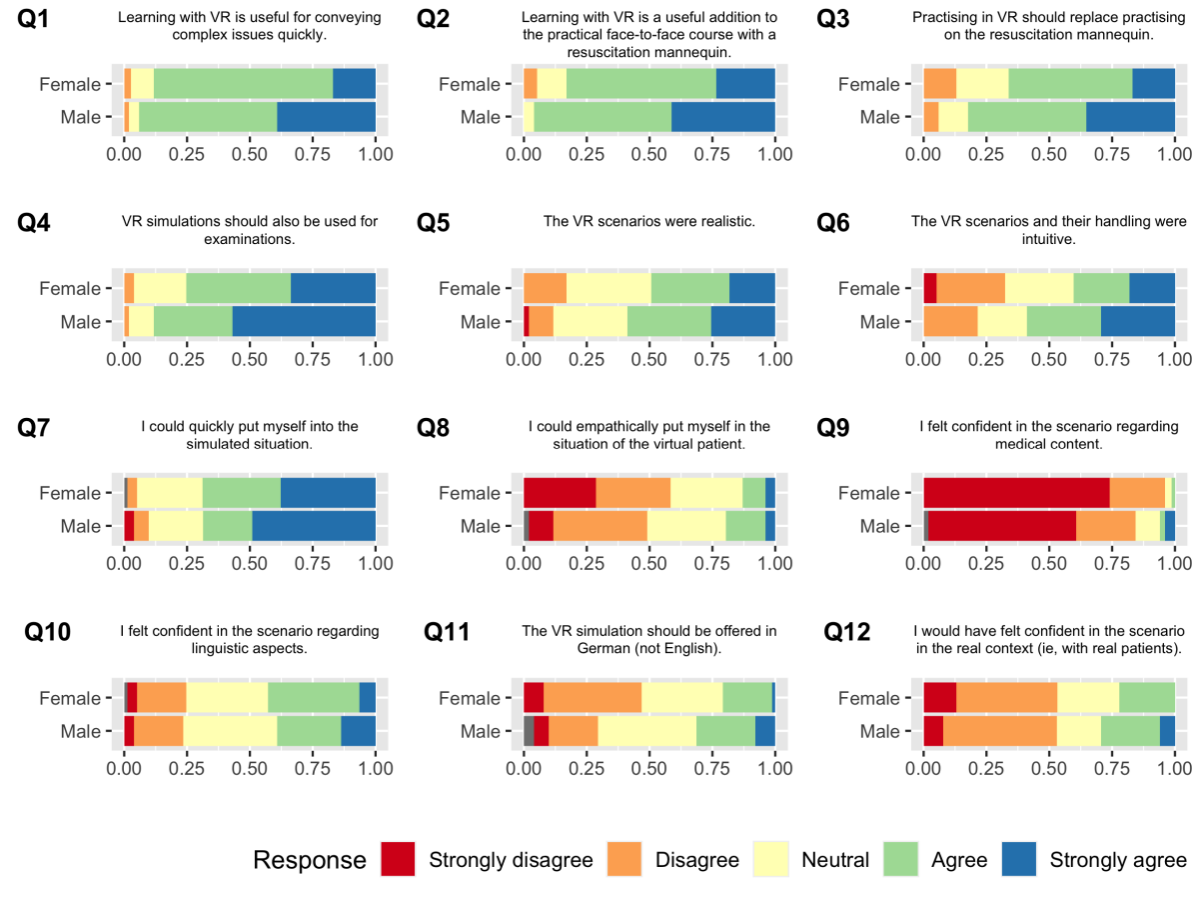
Answers to questions 1-13 (Q1-Q13) as a stacked bar chart, displayed for female and male participants separately. VR: virtual reality.

We further analyzed whether the students’ responses differed based on gender (Figure 2). For questions 1-4, female students showed a significantly stronger disagreement as compared to the male students (*P* values: Q1=.002; Q2=.005; Q3=.005; and Q4=.003). Moreover, a significantly lower number of female students agreed that the VR scenario and its handling were intuitive (Q6; *P*=.01). In addition, fewer female students stated that the VR simulation should be offered in German instead of English (Q11; *P*=.04). We did not detect any significant differences based on gender for the other questions.

### Impact of Previous EMS Experience on Questionnaire Answers

**Figure 3:**
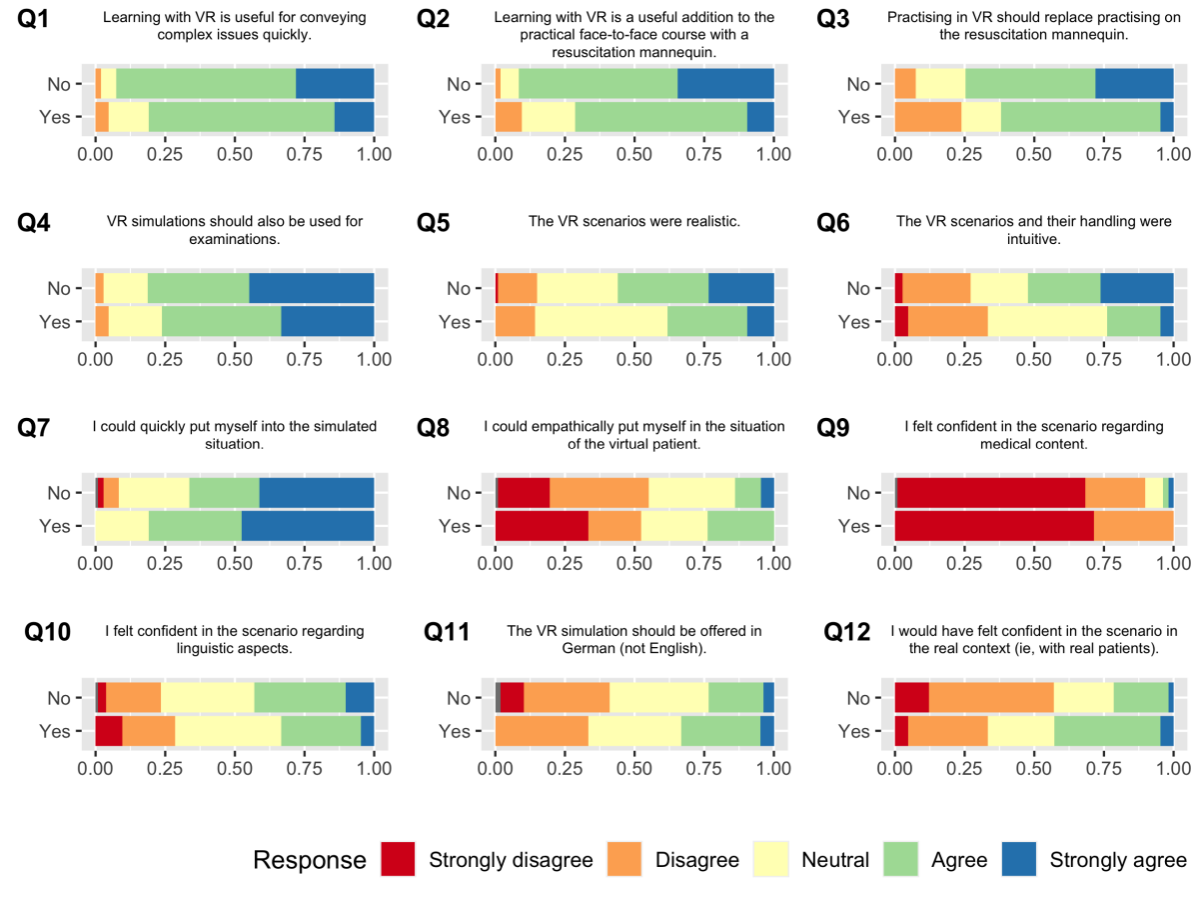
Answers to questions 1-13 (Q1-Q13) as a stacked bar chart, displayed for participants without and with previous experience in emergency medicine separately. VR: virtual reality.

We analyzed whether the responses to our questionnaire differed for students with and without prior EMS experience, that is, as paramedics (Figure 3). Students with previous EMS experience displayed a significantly lower agreement with the statements “Learning with VR is useful for conveying complex issues quickly” (*P*=.049), “Learning with VR is a useful addition to the practical face-to-face course with a resuscitation mannequin” (*P*=.001), and “Practising in VR should replace practising on the resuscitation mannequin” (*P*=.01). Students with prior EMS experience agreed significantly with Q12, which stated that they “would have felt confident in the scenario in the real context (ie, with real patients)” (*P*=.04). No significant differences were detected for the other questions.

### Physical Symptoms During VR Training

**Figure 4:**
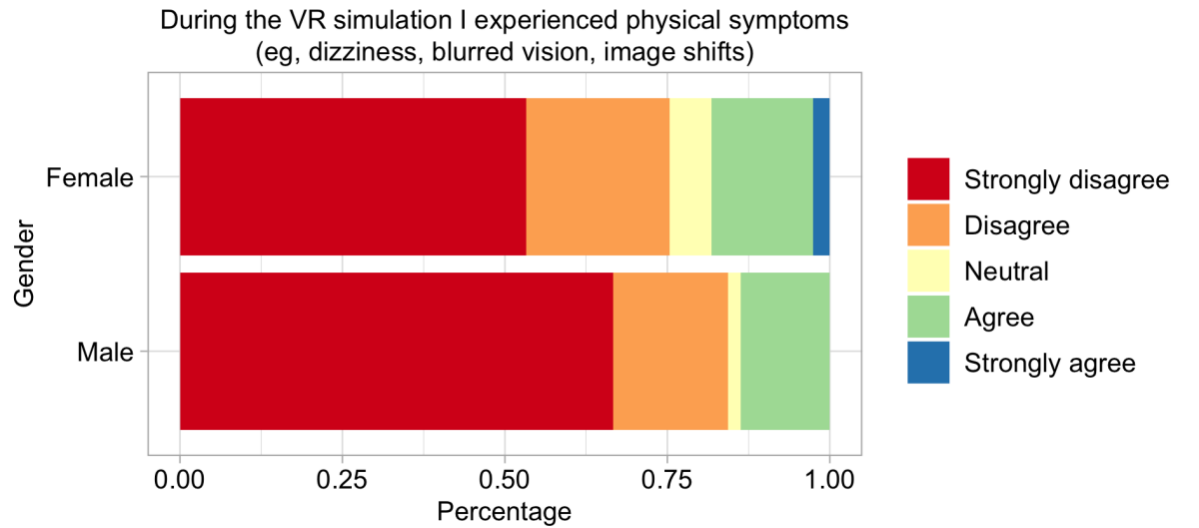
Symptoms by gender during the virtual reality (VR) simulation.

Of the respondents, 17.8% (n=23) experienced some side effects, with dizziness being the most frequent (n=10, 7.8%), followed by nausea (n=5, 3.9%), headache or blurred vision (n=3, 2.3% each), and drowsiness or shortness of breath (n=1, 0.7% each). However, none of the symptoms led to the termination of the simulation. The ordinal regression analysis revealed that female participants tended to experience greater physical symptoms (Figure 4), although the difference was not statistically significant (*P*=.12).

### Test Score

We analysed whether gender, age, or previous EMS and VR experience had any effect on the test scores for the resuscitation scenario (Table 2). However, the analysis revealed that none of these fixed factors had a significant impact on the test scores.

**Table 2:** Linear mixed-effects analysis for the impact of gender, age, and previous emergency medical services (EMS) and virtual reality (VR) experience on the test scores. Italicized *P* values indicate significance.

Predictors	Estimates	95% CI	*P* value
Intercept	68.11	35.38 to 100.84	*<.001*
Gender (female)	1.03	–5.31 to 7.38	.75
Age	–0.16	–1.24 to 0.93	.78
Previous EMS experience (no)	3.04	–5.23 to 11.31	.47
Previous VR experience from gaming (no)	–10.55	–25.60 to 4.50	.17

## Discussion

### Principal Results

In this study, we assessed the perspectives of a large cohort of fourth-year medical students on VR-based learning in emergency medicine. Overall, the students displayed strong agreement with the statement that VR can be useful for complex issues and a valuable addition to practical, mannequin-based emergency medicine courses. This is consistent with results from previous studies, which explored VR-based training of resuscitation instructors who also perceived VR as a valuable learning tool [15].

### Curricular Integration

Interestingly, most of the students agreed that VR should replace traditional mannequin-based practice and assessment. These questions were largely related to the concept of curricular implementation of VR as a teaching or assessment tool. However, the perceptions of the participants contradict certain real-world outcomes. For example, a recent randomized trial showed a higher resuscitation no-flow time after VR-based training in comparison to traditional training [7]. This is especially important because a short no-flow time could translate into an improved likelihood of survival [16]. Thus, it is important to consider the students’ enthusiasm in light of well-established training methods. Consequently, the curriculum planner should identify the learning objectives that can be achieved by using VR technology and how these translate into real-world (“real patient”) outcomes (constructive alignment). Although VR might be well suited for disaster medicine scenarios with a focus on situational decision-making [17] or for training students in medical procedures such as laparoscopic cholecystectomy [18], prior research [7] serves to remind curriculum designers to ensure that the use of such technology does not result in worse patient outcomes. It must be noted that the respondents were not entirely confident in the medical content and its transferability toward real patients. As the VR scenarios cover topics that are critical to emergency medicine (acute coronary syndrome, sepsis, stroke, and exacerbated chronic obstructive pulmonary disease), these results highlight that the handling of these scenarios poses a challenge to fourth-year medical students.

### Opportunities of VR-Based Teaching

However, despite these limitations, VR-based training can add valuable experiences to emergency medicine teaching. Due to the technical and virtual nature of VR-based scenarios, learners can repeat these scenarios almost indefinitely. For our scenarios and the students who participated in our study, we found a very low confidence in the scenario regarding the medical content (Figure 1, Q9). This translates into a low self-efficacy and might implicate a worse performance in future tasks [19]. Simulators, and especially VR-based simulations, have the opportunity to increase self-efficacy through different ways; they provide experiences in a safe environment that allows failure, provide feedback, and allow for a high number of trials [20]. Thus, we would expect the confidence of our learners and thus their self-efficacy to increase when the scenario can be repeated multiple times.

Interestingly, 54% (n=69) of our participants stated that they were unable to put themselves empathically into the situation of the virtual patient. Although empathy can be taught using VR-based teaching [21], there is no data indicating if empathy itself is required for learning in a VR-based environment. Following the empathy-altruism hypothesis, empathy can be seen as a requirement for altruistic action [22] and might thus be a perquisite for learning in patient-centered cases. To experience empathy, a study by Shin et al [23] revealed that the experience of quality, presence, and flow are important [23]. For future VR-based scenarios, it might thus be important to consider these aspects when designing patient scenarios.

In the light of our data and the aspects mentioned above, many opportunities appear for how VR-based teaching can augment current training. Most importantly, the opportunity of endless repetition and the possibility of covering seldom scenarios are clear chances of VR-based teaching. Some of these scenarios might even evolve to replace traditional teaching; however, care must be taken that patient outcomes are not impacted by this replacement. As physical feedback is of importance, augmented reality applications might be able to bridge this gap.

### Impact of Gender on Subjective Measurements

Previous studies have suggested that the students’ gender might be important for educational outcomes [11] and that females might be more prone to simulation sickness [24]. Interestingly, female students were significantly more reserved than their male peers in expressing agreement when answering half of the questions in our questionnaire. We observed this phenomenon in the case of the perceived usefulness of VR (Q1 and Q2) as well as willingness to accept VR-based teaching as a replacement for traditional methods of training and assessment (Q3 and Q4). However, we did not find a significant difference in previous experience with VR between female and male participants. The findings of Felnhofer et al [11], who surveyed psychology students, are consistent with our results, showing that females tend to experience a reduced sense of presence in a VR environment when compared with their male peers. In contrast, a study that surveyed technology and management students showed that females can even be more enthusiastic about VR when compared to males [25].

### Gender and Physical Symptoms

To explain potential gender differences, it has been suggested that females are more likely than males to suffer from severe motion sickness during VR simulations [24]. Consistent with these findings, we observed a trend toward greater physical symptoms in females, although this difference was not statistically significant. Further, a recent review challenges this association [26]. These conflicting results could potentially be explained by differences in technical aspects. For example, a study by Stanney et al [27] demonstrated that an interpupillary distance nonfit happened significantly more often among females, which could explain why females were found to experience motion sickness more than males in some studies. Our findings also suggest that there might be significant differences between female and male students in terms of the usefulness of VR as a teaching tool. From the curriculum perspective, this difference should be kept in mind when traditional teaching is replaced by VR-based teaching.

### Limitations

This study has certain limitations. First, it was performed at 1 medical faculty in 1 country within a single teaching course, which detracts from its generalizability. Second, our VR unit was limited to the hardware and software of 1 manufacturer each. However, these represent state-of-the-art products, thus reflecting the current status of VR development. Moreover, the scenario was rated as realistic and the handling as intuitive by the students. Third, as we were interested in curricular aspects of VR-based teaching, we used a self-developed questionnaire to cover our research interest within one questionnaire. Influenced by the burdens of the COVID-19 pandemic, our intention was to limit the efforts our students had to take for our study, thus enabling more time for practical training. However, in future investigations, additional validated questionnaires should be used. Furthermore, due to the aforementioned aspects, we only interviewed our students after the VR session. This enabled us to study a large number of students due to the limited efforts necessary for participating; however, we cannot assess how the VR experience influenced certain aspects, that is, the confidence of our learners before and after the scenario. In future studies, a strong link between training and assessment as well as virtual and face-to-face settings should be considered.

### Conclusions

In summary, in this large-scale assessment, we showed that medical students are largely enthusiastic towards VR-based teaching and assessment. Although these traits were also found among female students, they were more reserved when compared to male students. Thus, before VR is implemented in the medical curriculum, special attention needs to be focused on designing the relevant courses so that every student can benefit from these interventions; this could potentially be achieved by overcoming technical restrictions. In general, although students are positive about the replacement of traditional teaching methods with VR-based training and it is seen as a useful supplementary tool for medical education, care should be taken to ensure that patients’ outcomes do not worsen as a result.

## Abbreviations

CPR: cardiopulmonary resuscitation
EMS: emergency medical services
Q: question
VR: virtual reality
